# 

**Published:** 1840-10

**Authors:** 


### CONTENTS OF No. XX.

OF THE

### British and Foreign Medical Review.

OCTOBER, 1840.

### Part First.—Analytical and Critical Reviews.

PAGE

Art. I.—1. Cases and Observations, illustrative of Renal Disease, accompanied with the Secretion of Albuminous Urine. By Dr. Bright . . 301
2. De l'Albuminurie ou Hydropisie causée par Maladie des Reins, &c. Par le Dr. Martin-Solon . . . . . . . ib.
On Albuminuria or Dropsy caused by Diseased Kidney. By M. Solon, m.d.
3. Traité des Maladies des Reins et des Altérations de la Sécrétion Urinaire, &c. Par P. Rayer . . . . . . . ib.
Treatise on Diseases of the Kidneys, &c. By P. Rayer, m.d. &c.
4. On Granular Degeneration of the Kidneys, and its Connexion with Dropsy, Inflammations, and other Diseases. By Robert Christison, m.d., f.r.s.e. ib.
5. Observations on Abdominal Tumours and Intumescence; illustrated by Cases of Renal Disease. By Dr. Bright . . . . ib.
6. Cases and Observations illustrative of Renal Disease, accompanied with the Secretion of Albuminous Urine. Memoir the Second. By Dr. Bright . ib.
7. Cases of Albuminous Urine, &c. By Dr. G. H. Barlow . . ib.

Art. II.—1. Pathological and Surgical Observations on Diseases of the Joints. By B. C. Brodie, v.p.r.s., &c. . . . . . 330
2. The Cyclopædia of Anatomy and Physiology, July—Articles—Elbow, Joint of the; Abnormal Anatomy—Hip-joint, Abnormal Conditions of; Knee-joint, Abnormal Conditions of. By R. Adams, Esq. . . . ib.
3. Cyclopædia of Practical Surgery. Art. Articulations. By J. Blackburn, Esq. ib.
4. Dissertatio Medica Inauguralis de Cartilaginum Articularium ex Morbis Mutatione. Auctore L. H. Schumer, Jun. . . . . ib.

Art. III.—1. Traité complet d'Anatomie Chirurgicale, Générale et Topographique, du Corps Humain. Par Alf. A. L. M. Velpeau . . . 347
A complete Treatise on the Surgical Anatomy of the Human Body, both general and regional. By Alf. A. L. M. Velpeau.
2. Traité d'Anatomie Chirurgicale et de Chirurgie Expérimentale. Par J. F. Malgaigne . . . . . . . ib.
A Treatise on Surgical Anatomy and Experimental Surgery. By J. F. Malgaigne.

Art. IV.—Ueber den Einfluss der verschiedenen Gewerbe auf dem Gesundheitszustand und die Mortalität der Küntsler und Handwerker in dem Blüthenjahren, &c. Von Dr. C. H. Fuchs . . . . 373
A Statistical Memoir on the Influence of various Professions on the Health and Mortality of Mechanics and Artisans in the prime of life; founded on the Tables of the Institution for Sick Mechanics in Würtzburg from 1786 to 1834. By Dr. C. H. Fuchs.

Art. V.—1. Traité pratique des Maladies Vénériennes. Par Ph. Ricord . 381
A Practical Treatise on Venereal Diseases. By Ph. Ricord.
2. Recherches pratiques sur la Thérapeutique de la Syphilis, etc. Par Lucas Championniere, m.d. . . . . . . ib.
Practical Researches on the Therapeutics of Syphilis, &c. By L. Championniere.
3. A Treatise on the Venereal Disease and its Varieties. By Wm. Wallace . ib.
4. A Treatise on Syphilis. By Herbert Mayo, f.r.s. . . . ib.
5. The Modern Treatment of Syphilitic Diseases, both primary and secondary, containing an account of the new Remedies. By Langston Parker . ib.
6. Report of Primary Syphilitic Cases. By C. Aston Key . . ib.
7. On the Venereal Cases in which Mercury is admissible or is not admissible.—Clinical Lectures on the Venereal Disease. By Richard Carmichael . ib.

§

PAGE

8. Manuel Pratique de la Maladies de la Peau, appelées Syphilides, d'après les Leçons Cliniques de M. Biett. Par Jos. HUMBERT, D.M.P. . . ib.
A Practical Manual of the Venereal Diseases of the Skin, from the Clinical Lectures of M. Biett. By Jos. HUMBERT, D.M.P.
9. A Treatise on the Venereal Diseases of the Eye. By WM. LAWRENCE, F.R.S. ib.
10. A Practical Treatise on the Diseases of the Eye. By WM. MACKENZIE . ib.
11. Observations on the Venereal Disease and Venereal Inoculation. By W. ACTON ib.
12. Beiträge zur Lehre von den Syphilitischen Krankheiten. Von Dr. HAUCK . ib.
Contributions to our Knowledge of Syphilitic Diseases. By Dr. HAUCK.

ART. VI.—1. Statistica delle Morti Improvvise, e particolarmente delle Morti per Apoplessia, nella Citta e nel Circondario Esterno di Milano, dall' anno 1750 al 1834. Del GIUSEPPE FERRARIO, D.M. . . . . 420
Statistics of Sudden Deaths, more particularly of Deaths from Apoplexy, in the city and neighbourhood of Milan, from 1750 to 1834. By J. FERRARIO, M.D.
2. Monografia sulle Morti Repentine. Del Signor N. M. SORMANI, D.M. . ib.
Monography on Sudden Deaths. By N. M. SORMANI, M.D.

ART. VII.—Traité des Maladies de Plomb ou Saturnines. Par L. TANQUEREL DES PLANCHES, M.D. . . . . . . 426
Treatise on the Diseases occasioned by Lead. By L. TANQUEREL DES PLANCHES, M.D.

ART. VIII.—Transactions of the Provincial Medical and Surgical Association . 461

ART. IX.—Crania Americana; or a Comparative View of the Skulls of various Aboriginal Nations of North and South America; with an Essay on the Varieties of the Human Species. By S. G. MORTON, M.D. Woodcuts . 474

ART. X.—1. Histoire complète des Ruptures et des Dechirures de l'Uterus, du Vagin, et du Périnée. Par F. DUPARCQUE . . . . 486
A complete History of Ruptures and Lacerations of the Uterus, Vagina, and Perineum. By F. DUPARCQUE.
2. F. Duparcque's Vollstandige Geschichte der Durchlöcherungen, Einrisse, und Zereissungen des Uterus, &c. Von J. F. W. NEVERMANN, M.D. . ib.
F. Duparcque's complete History of Perforations, Lacerations, and Ruptures of the Uterus, &c. By J. F. W. NEVERMANN, M.D.

ART. XI.—Anatomical, Pathological, and Therapeutic Researches on the Yellow Fever of Gibraltar of 1828. By P. C. H. A. LOUIS. Trans. by Dr. SHATTUCK 494

ART. XII.—Comparative Anatomy of the Nervous System. By JOSEPH SWAN . 508

ART. XIII.—Physiological and Moral Management of Infancy. By Dr. A. COMBE 514

ART. XIV.—An Essay on the Treatment of some Affections of the Prostate Gland. By R. A. STAFFORD . . . . . . 528

ART. XV.—First Principles of Surgery, being an Outline of Inflammation and its Effects. By GEORGE T. MORGAN, A.M. . . . . 532

ART XVI.—A System of Practical Surgery, with numerous explanatory plates and drawings after nature. By JOHN LIZARS . . . . 537

ART. XVII.—Supplement to the Introduction to the Atomic Theory. Prefaced by some Remarks on the projected Reforms in Academical Education. By CHARLES DAUBENY, M.D., F.R.S., &c. &c. . . . . 542

ART. XVIII.—Observations on the Diseases incident to Pregnancy and Childbed. By FLEETWOOD CHURCHILL, M.D., &c. . . . . 544

### PART SECOND.—Bibliographical Notices.

ART. I.—Vital Dynamics; the Hunterian Oration before the Royal College of Surgeons in London, 14th February, 1840. By JOSEPH H. GREEN, F.R.S., &c. 545

ART. II.—The Anatomist's Vade Mecum. By W. J. ERASMUS WILSON . 547

ART. III.—Practical Observations on Abortion. By J. S. STREETER . . 548

ART. IV.—An Essay on the Treatment and Cure of Pulmonary Consumption, on principles natural, rational, and successful. By GEORGE BODDINGTON . 549

ART. V.—Forhandlingar vid det af Skandinaviska Naturforskare och Läkare, &c. ib.
Transactions of the Scandinavian Society of Naturalists and Physicians, 1839.

PAGE

ART. VI.—Microscopic Illustrations of Living Objects. By C. R. GORING, M.D., and A. PRITCHARD . . . . . . 550

ART. VII.—A Practical Essay on the Diseases generally known under the denomination of Delirium Tremens. By ANDREW BLAKE, M.D. . . ib.

---

### PART THIRD.—Selections from the British and Foreign Journals.

### I. THE FOREIGN JOURNALS.

#### ANATOMY AND PHYSIOLOGY.

Dr. HENLE on the Contractility of the Blood-vessels . . . 551
Professor NASSE on the Alterations which Nervous Fibres undergo after Division . 553
M. MONAT'S Experimental Researches on the Functions of the Encephalon . 554
Dr. DE GRAVINA'S Philosophical and Experimental Researches into the True and Primary Action of the Secale Cornutum as a Medicine . . . ib.

#### PATHOLOGY, PRACTICAL MEDICINE, AND THERAPEUTICS.

Dr. HOFFMAN on Cramp of the Tongue . . . . . 557
Dr. GROSSHEIM on the Pain of the Back in Intermittent Fever . . ib.
GMELIN on the Presence of Iodine in Cod-liver Oil . . . 558
Dr. ASCHERSON on the Rest-harrow, a Remedy for Rheumatism . . . ib.
M. BARRIER on the Production of Chronic Ventricular Hydrocephalus . . 559
Dr. BRUNZLOW'S Coincident Occurrence of Variola, Modified Variola, and Varicella 559
Dr. PETERSEN'S Case of Menstruatio Recidiva . . . 560
M. BECQUEREL'S Anatomico-Pathological Researches on Cirrhosis of the Liver . ib.
M. PUCHE on the Danger of applying Leeches which have been previously used . 563
Dr. KRAUSE on the Itch in Adults and in Children . . . . 564
MM. GELIS and CONTE on the Employment of Lactate of Iron . . 565

#### SURGERY.

M. LAUGIER on a New Species of Strangulation in the Tunica Vaginalis Testis followed by Internal Strangulation . . . . 565
M. HOURMANN on Peritonitis from Injections into the Uterus, the Injections passing through the Fallopian tubes into the Cavity of the Peritoneum . . 566
M. GAILLARD on Dislocation of the Humerus reduced after Sixteen Years . ib.
Professor BERTHOLD on the Myopodiorthoticon, or Apparatus for the Cure of Shortsightedness . . . . . . . 567
M. GUERIN on Luxation of the Second Cervical Vertebra, . . . ib.
M. ROBERT'S New Operation for Prolapsus Ani . . . . 568
Dr. ARONSOHN'S communication of Two Cases of the Excision of the entire Clavicle ib.
M. BIAGINI on the Radical Cure of Inguinal Hernia by the Horizontal Position . 569
Professor DIEFFENBACH on the operation for Strabismus . . 570
M. JULES GUERIN—his Claims on the Operation for Strabismus . . 572
Dr. VON AMMON on the Cure of Strabismus . . . . 573
M. CIVIALE on certain Abscesses occurring in different parts of the Body, during the course of Diseases of the Urinary Organs . . . . 574
M. JOBERT'S New Operation for the Cure of Vaginal Cystocele . . 575
Dr. GUERIN on the Etiology and Treatment of Luxations of the Femur . 576
M. JOBERT'S Therapeutic Considerations on Obliterations of the Veins in the Treatment of Varix and Varicose Ulcers . . . . 577

#### MIDWIFERY.

Dr. LÖWENHARDT on Fracture of the Upper Arm of a Child during Birth . ib.
Dr. GODFROY'S Successful Case of Cæsarian Section, with Suture of the Uterus . 578
Dr. LÖWENHARDT'S Case of Puerperal Fever, with unusual Sloughing . . 579
Dr. SCHÖLLER on Phlebitis Hepatica Neonatorum . . . . 580

#### MEDICAL JURISPRUDENCE AND TOXICOLOGY.

Dr. LOHMEIER on Poisoning by Antimonial Vapours . . . 580

PAGE

### CHEMISTRY.

M. Borsarelli's Mode of discovering Adulteration of Essential Oils with Alcohol 582

### STATISTICS.

Longevity in Russia . . . . . . 582

Mortality of St. Petersburg . . . . . . ib.

Danish Medical Statistics . . . . . . 583

### II. THE BRITISH JOURNALS.

Dr. Smith's Observations on the Diseases of Peru . . . . 583
Dr. Peebles's Cases of Great Enlargement of the Stomach, with Remarks . ib.
Mr. Thomson's Cases of Deformity of the Feet cured by the Operation of Stromeyer ib.
Dr. Gardner on Anomalous Affections of the Respiratory Organs . . . ib.
Dr. Burgess on Nervous Headach from Exhaustion . . . 584
Dr. Maclagan on the Action of Hydrated Sesquioxide of Iron on Arsenic . ib.
Mr. Donovan on Cod-oil . . . . . . ib.
Mr. Hamilton on the Utilitiy of Oxymuriate of Mercury in Strumous Ophthalmia ib.
Mr. Donovan on the Hydrocyanoferrate of Quinina . . . ib.
Dr. Ferguson's Direct Application of Galvanism to the Diaphragm . . ib.
Dr. O'Beirne on real and apparent Enlargement of the Eyeball . . 585
Mr. Lucas on Division of the Internal Rectus Muscle in Cases of Squinting . ib.
Mr. Ancram on the Causes and Treatment of Strabismus . . . ib.
Mr. Barlow's Case of Hemiplegia . . . . . ib.
Mr. Obre on Gangrene of the Mouth successfully treated by the Actual Cautery . ib.
Mr. Lightfoot's Three Cases of Strabismus cured by Operation . . ib.
Mr. Lietch's New Mode of Operating for the Cure of Squinting . . ib.
Mr. Lucas's Cases of Strabismus cured by Operation; with Observations . ib.
Mr. French on Abandonment of the Hook in the Operation for Squinting . 586
Mr. Adams's New Hook for Dividing the Internul Rectus Muscle of the Eye . ib.
Dr. Munk on the Use of Sulphur in some Spasmodic Affections . . ib.
Mr. Clay on the Use of Sulphur in Rheumatic Affections . . . ib.
Mr. Mackmurdo on the Operation for Squinting . . . . ib.
Mr. Adams on a New Form of Sharp Hook for fixing the Eye during the Operation ib.
On Deafness and Dumbness produced by the Use of Quinine . . ib.
Dr. Bird's Observations on the Occurrence of Cerebral Disorders . . ib.
Dr. Lee on the Situation of the Deciduous Membrane in Extra-uterine Gestation 587
Mr. Curling's Case of Sero-cystic Tumours of the Breast removed by Operation . ib.
Mr. Mayo on the Effects of Dividing the Inner Straight Muscle of the Eye . ib.
Mr. Thomson on the Influence of Woollen Manufactures on Health . . ib.
Dr. Scott on Dividing the Internus Rectus Muscle for the Cure of Strabismus . ib.
Dr. Franz's Cases of Strabismus . . . . . ib.
Dr. Durrant on Congenital Peculiarity in the Structure of the Heart . . ib.
Dr. Gregory on the Statistics of Smallpox . . . 588
Dr. Hannay on the Treatment of Croup . . . . . ib.
Dr. Lawrie's Cases illustrative of the Poisonous and Injurious Effects of the Hydriodate of Potash and Iodide of Starch . . . . ib.
Dr. Birt's Curious Case of Pin Swallowing . . . . ib.
Dr. Franz's Two Cases of Squinting . . . . . ib.
Dr. Pidduck on the Use of Strychnine in Neuralgia . . . ib.
Mr. French on the Operation for Strabismus . . . . ib.

### Part Fourth.—Medical Intelligence.

New Bills of Mortality for London . . . . 589

The Thackeray Prize . . . . . 591

The Respirator . . . . . . . ib.

Dr. Lee on the Function of the Ovaries in Menstruation . . . 592

Dr. Bushnan's Observations on Dysmenorrhœa . . . 593

OBITUARY—Drs. Sanden and Todd . . . . 596

Books received for Review . . . . 600

Title, Index, &c.

